# Prenatal and Intrapartum Factors Associated With Infant Temperament: A Systematic Review

**DOI:** 10.3389/fpsyt.2021.609020

**Published:** 2021-04-08

**Authors:** Mizuki Takegata, Asami Matsunaga, Yukiko Ohashi, Michiko Toizumi, Lay Myint Yoshida, Toshinori Kitamura

**Affiliations:** ^1^Department of Pediatric Infectious Diseases, Institute of Tropical Medicine, Nagasaki University, Nagasaki, Japan; ^2^Kitamura Institute of Mental Health Tokyo, Tokyo, Japan; ^3^Kitamura KOKORO Clinic Mental Health, Tokyo, Japan; ^4^Department of Community Mental Health and Law, National Institute of Mental Health, National Center of Neurology and Psychiatry, Kodaira, Japan; ^5^Faculty of Nursing, Josai International University, Togane, Japan; ^6^T. and F. Kitamura Foundation for Studies and Skill Advancement in Mental Health, Tokyo, Japan; ^7^Department of Psychiatry, Graduate School of Medicine, Nagoya University, Nagoya, Japan

**Keywords:** infant, temperament, systematic review, prenatal factor, intrapartum factor

## Abstract

**Background:** Temperament involves individual variations in behavioural tendencies of emotional responses and reactions to stimuli after birth. Because ‘foetal programming' is a strong hypothesis in developing temperament, prenatal and intrapartum factors may be significant determinants of infant temperament. This systematic literature review aims to elucidate the evidence of prenatal and intrapartum predictors, including genetic, biological, environmental, socio-demographic, psychological, and obstetric factors of parents and their child.

**Methods:** Relevant articles were searched using MEDLINE, PubMed, and SCOPUS. The inclusion criteria were (a) original research article, (b) written in English, (c) assessed the temperament of infants 12 months old or younger as an outcome variable, and (d) investigated prenatal and intrapartum factorial variables of infant temperament. Following the PRISMA guideline, the articles found in the three databases were screened and selected according to the inclusion and exclusion criteria before the final review.

**Results:** Finally, 35 articles were reviewed. This systematic review identified a variety of prenatal and intrapartum factors that were significantly associated with infant temperament: (1) genetic and biological factors: certain genotypes, maternal cortisol and ACTH, and CRHs, (2) environmental factors: substance use such as tobacco, alcohol, and illegal drugs, (3) socio-demographic factor: lower-income, (4) psychological factors: depression or anxiety, eating disorders, personality types of mothers, and domestic violence, and (5) obstetric factors: foetal growth (birth weight), hypertension in mothers, nausea (emesis), and preterm birth.

**Conclusion:** The findings support gene-environment interaction and biological mechanisms for developing infant temperament, suggesting the importance of ensuring a safe and comfortable environment for pregnant mothers, unborn infants, and families during pregnancy and delivery.

## Introduction

In psychiatry, temperament involves individual variations in behavioural tendencies of emotional responses and reactions to stimuli after birth (1). Different concepts and classifications for temperament have been developed. For example, the temperament is conceptualised as ‘the stylistic component of behaviour' (2) showing nine behavioural styles (e.g., activity and regularity). These styles were further categorised into three types of children in terms of how they fit into the family and school: easy, difficult, and slow-to-warm-up (2). Following Chess and Thomas's theory, Buss and Plomin (3) defined temperament as ‘a set of inherited personality traits that appear early in life' (3). Rothbart defined temperament as ‘relatively stable, primarily biologically based individual differences in reactivity and self-regulation' (4). These researchers indicated that temperament developed before higher cognitive and social aspects of personality, and labelled three major dimensions of temperament: surgency/extraversion, negative affect, and effortful control (5). Hence, temperament is considered to be biologically based, exists from early infancy, and plays a core role in forming an individual's personality (1). Some researcher suggested that, although temperament was originally viewed as relatively stable across an individual's life course, the *expression of* temperament may be modifiable following the interaction with external environments (1).

Chess and Thomas (2) first proposed the difficult temperament of a child. The difficult temperament refers to a child who tends to react negatively to new situations, is fussy, irritable, and may show other negative reactions (2). Their research placed ~40% of American children in the easy child group, 10% were categorised as difficult, and 15% as slow to warm up (2). Several studies have reported that difficult temperament predisposes the child to emotional difficulties, hyperactivity/inattention, and behaviour problems in later childhood and adolescence (6–8). Although the mechanism is not clear, temperament may act as a mediator or moderator contributing to behaviour problems later in life. For example, when a child comes across a stranger, a difficult temperament child would react negatively, such as crying or throwing a tantrum, whereas others may show positive reactions, such as smiling and laughing. Hence, subjective experiences vary among children depending on their reactivity and self-regulation patterns (9).

Although it is generally believed that temperament is formed from biological reactions before birth, as stated above, its mechanism is a mystery. First, ‘foetal programming' is a strong hypothesis of neuropsychological and behavioural development (10). Genetic factors are thought to play an independent role (11) or interact with environmental factors (12–15). Additionally, the programming is influenced by the neurobiological stress-response system of the child, including immune, cardiovascular, and the hypothalamic-pituitary-adrenal axis (HPA) systems exposed to external stressors (16–18). Second, ‘biological embedding' is another hypothesised mechanism. Biological embedding refers to ‘the process by which early life experiences alter biological processes to affect adult health outcomes' (19) and starts in early pregnancy in response to environmental stressors, bringing structural and functional changes at both molecular and physiological levels (12, 20, 21). Hence, prenatal environmental factors should be emphasised to understand the development of a child's temperament (22, 24).

To the best of our knowledge, four relevant systematic reviews have been conducted on the relationship between prenatal mental disorders in mothers and infant temperament. Two reviews, published in 2011 (22) and 2017 (23), found that prenatal depression and anxiety in the mother was associated with the difficult temperament of her child. However, another review published in 2017 (24) concluded that the association was equivocal due to the limited number of articles. Furthermore, another review found that prenatal alcohol consumption by the mother predicted lower positive affect, affiliation/regulation, and orienting capacity of the infant (25). Besides these reviews, one study determined that increased DNA methylation gestational age relative to clinical gestational age was associated with low-socioeconomic status and birth weight independent of gestational age, sex, and ancestry (26), indicating that prenatal stress due to low socioeconomic status may influence foetal programming. In addition, attention is increasingly being paid to the effect of nutritional supplements, such as omega-3 (*n* = 3) docosahexanoic acid (DHA), on cognitive and neurological development (27, 28). Therefore, considering foetal programming and biological embedding as the possible mechanisms, prenatal factors of temperament may include not only the psychological distress of mothers but also genetic, biological, and socio-demographic aspects such as economic status, maternal nutrition, and pregnancy and delivery complications.

This systematic literature review elucidates the evidence of prenatal and intrapartum predictors, including genetic, biological, socio-demographic, psychological, and obstetric factors of parents and their infant.

## Methods

Appendix 1 shows the search strategy for this literature review. Relevant articles were searched by using MEDLINE, PubMed, and SCOPUS. The search terms were as follows: ‘perinatal OR antenatal OR pregnancy,' ‘mental stress OR psychological stress OR depress^*^ OR anxiety OR posttraumatic stress,^*^' ‘factor OR predictor OR determinant,' ‘infant,' and ‘temperament.' Inclusion criteria were (a) original research article, (b) written in English, (c) assessed temperament of infants who were 12 months old or younger as an outcome variable, and (d) investigated prenatal and intrapartum factorial variables of infant temperament. We limited our search to infancy because it is believed that the expression of temperament is relatively stable in infancy (1). A cross-sectional article that assessed dysphoric moods perceived by parents along with infant temperament during the postpartum period was excluded because emotionally impaired mothers are reportedly more likely to report their child as having behavioural problems (29). Following the PRISMA flow diagram, relevant articles were screened on the three databases. Titles and their abstract of records, after removing duplicates, were reviewed using ENDNOTE software by M.T. (1^st^ author). Full-text articles of relevant records were carefully reviewed by M.T. (1^st^ author) and A.M. (2^nd^ author) according to the inclusion and exclusion criteria and the quality of each article was assessed. After a discussion, 35 articles were selected for the review.

## Results

**Figure 1:**
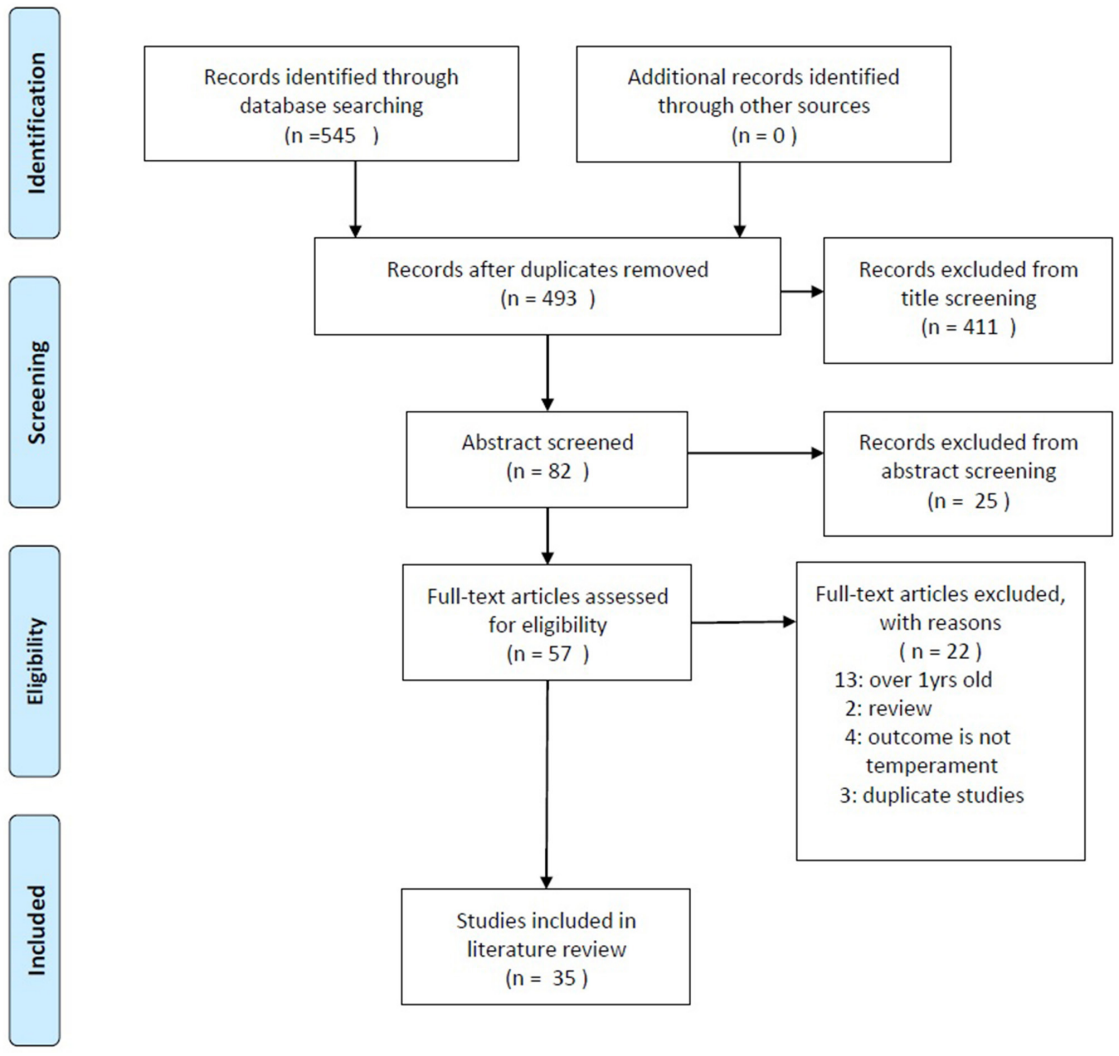
Flow diagram of search strategy.

Figure 1 shows a flow chart of the search strategy. Of the 545 papers found on the databases as of 11 March 2020, title screening yielded 82 (15.0%) original articles. Next, 57 articles (10.4%) were selected through title and abstract screening. Finally, after a discussion between the authors, 35 papers (6.4%) were included in the final review.

Nineteen papers (3.4%) were excluded because they did not meet the inclusion criteria: (a) 13 studies observed the temperament of infants older than 12 months of age, (b) two studies were identified as literature reviews, (c) four studies assessed infants' neuro-motor development but not temperament, and (d) two articles referred to the same study as one of the included articles and one did not assess predictors of a child's temperament. Appendix 2 and Appendix 3 summarise the final list of articles and quality assessment, respectively.

### Research Trends

Table 1 shows the characteristics of the studies finally reviewed, including region, year published, study design, assessment point of infant temperament, and methods of assessing infant temperament. Most of the studies were conducted in Europe (*n* = 15) or North America (*n* = 17), whereas only a few were conducted in Oceania (*n* = 2) and Asia (*n* = 1). It was also evident that the number of articles published in this field had increased dramatically after 2006, although this research theme has been emphasised since 1978 (30). Regarding the study design, only a few studies conducted a cohort design (*n* = 10) or assessed infant temperament during the neonatal period (*n* = 2). Regarding the methodologies of assessing infant temperament, all the studies used parent-administrative questionnaires; however, two studies adopted the observational assessment of child behaviour by clinical specialist (*n* = 3). The most frequently used questionnaire was the Infant Behaviour Questionnaire (IBQ), its revised version (31) or a very short version (32) (*n* =13), followed by the Infant Characteristic Questionnaire (ICQ) (33) (*n* = 6), the Carey Infant Temperament Questionnaire (CITQ) (*n* = 4) (34), and the Thomas and Chess Temperament Questionnaire (TTQ) (*n* = 2) (35), among others. As for the observation of infant behaviour, Kagan's temperament scores were used to observe infants' reactivity in a strange situation (36), as well as *ad-hoc* methods.

**Table 1:** Study characteristics of included.

**Study characteristics**	***N*^**1**^**	**Article *N*^**2**^**
**Region**		
Europe	15	4, 6, 7, 11, 14, 15, 16, 17, 19, 21, 28, 29, 31, 33, 35
North America	17	1, 3, 5, 8, 9, 10, 12, 18, 20, 21, 23, 24, 25, 27, 30, 32, 34
Oceania	2	13, 26
Asia	1	2
**Year published**		
2016–2019	5	1–5
2011–2015	10	6–15
2006–2010	9	16–24
2001–2005	6	25–30
1996–2000	1	31
1991–1995	0	0
Before 1990	4	32–35
**Study design**		
Cohort study	10	5, 8, 13, 15, 19, 20, 22, 30, 33, 34
**Observational point of assessing temperament**		
Neonatal period	2	18, 32
2 month to 5 month of age	13	2, 5, 9, 10, 11, 13, 16, 17, 21, 23, 26, 27, 29
6 month to 11 month of age	17	1, 3, 4, 6, 7, 8, 15, 19, 20, 22, 23, 25, 26, 28, 31, 33, 35
12 month of age	4	12, 13, 30, 34
**Measure assessing infant temperament**		
**Parent administered questionnaire**		
Infant Behavior Questionnaire (IBQ)	13	1, 4, 5, 6, 7, 8, 9, 10, 11, 19, 21, 23, 27
Infant Characteristic Questionnaire (ICQ)	6	3, 15, 22, 28, 29, 30
Thomas and Chess's temperament questionnaire (TTQ)	2	33, 35
Carey Infant Temperament Questionnaire (CITQ)	4	12, 20, 25, 31
Others (including *ad hoc* questions)	9	2, 13, 14, 16, 17, 18, 24, 26, 32
Observational assessment of infant behaviour	3	5, 23, 29

*1. Number: Number of relevant articles 2. Article Number: Article number referred to Appendix 2*.

### Prenatal and Intrapartum Factors for Infant Temperament

Table 2 summarises the results of prenatal and intrapartum factors for infant temperament.

**Table 2:** Findings of literature review.

**Prenatal factor**				**N^**1**^**	**Article N^**2**^**	**Summary finding**
**1**.	**Genetic and biological factors**	**1.1**.	**Genes**			
		1.1.1.	Genetic factors (compared Monozygotic and Dizygotic twins)	1	35	Monozygotic twins were more similar in temperament scores than dizygotic twins, indicating that genetic factors may play a role in temperament.
		1.1.2.	5HTT, DRD4, and MAO-A	2	17, 18	Genes-environment interaction between *TaqIA* polymorphism, 5-HTTLPR genes, tobacco intake, and prenatal anxiety among mothers was related to higher attention and irritability in the infant.
		**1.2**.	**Biological factors**			
		1.2.1.	Maternal cortisol (saliva or blood), placental cortisol, Corticotropin-releasing hormone (CRH) or Adrenocorticotropic hormone (ACTH)	4	10, 21, 27, 29	Most of the studies reported that a significant correlation between increased levels of maternal stress hormone during pregnancy and higher difficult temperament of infants, such as higher emotional activity, higher irritability and fussiness and higher fear and distress feelings.
		1.2.2.	Fetal heart rate	1	23	Third trimester foetuses who showed heart rate increases exposed to maternal stress were more likely to exhibit high motor reactivity in response to novelty at 4 months of age.
**2**.	**Environmental**	**2.1**.	**Substance use**			
	**factors**	2.1.1.	Smoking	3	18, 20, 25	Prenatal smoking was associated with lower positive mood and lower reactivity to stimuli, higher attention and irritability among infants.
		2.1.2.	Alcohol (Maternal or Paternal)	1	30	Infants of an alcoholic father displayed ‘stubborn' and ‘persistent temperaments' at 12 months of age.
		2.1.3.	Illegal drug	2	5, 24	Infants of mothers who used cocaine or opiate scored lower on ‘smiling and laughter', or ‘poor attention to stimuli'.
**3**.	**Socio-demographic factors**	**3.1**.	**Low income/ethnic minority**	2	1, 8	Prenatal stress caused by stressful events among low income women predicted lower ‘surgency' and ‘regulation' of infants.
		**3.2**.	**Maternal nutrition**			
		3.2.1.	Chocolate consumption	1	28	No significant relationship between chocolate consumption and infant temperament.
		3.2.2.	Intakes of polyunsaturated fatty acids (PUFAs) (n3, n6)	1	8	Prenatal stress effects on infant ‘orienting' & ‘regulation' were modified by maternal n3: n6 ratios among African American.
**4**.	**Psychological factors**	**4.1**.	**Maternal mental health**			
		4.1.1.	Prenatal depression or anxiety among mothers	9	2, 4, 6, 10, 11, 13, 14, 26, 32	Both prenatal depression and anxiety among mothers were associated with difficult temperaments, whereas some denied the causal relationships. A variety of infant temperaments were moderately or weakly associated with the dysphoric moods such as negative affectivity, emotionality, activity, fussiness, poor attention, and fearfulness.
		4.1.2.	Psychological stress	5	1, 3, 8, 9, 27	Prenatal psychological stress due to severe stressful events predicted lower or higher surgency, regulation, fussiness, dullness and poor attention.
		4.1.3.	Mindfulness	1	7	Maternal mindfulness is negatively related to difficult temperament of the child.
		4.1.4.	Eating disorder	1	15	Infants of mothers diagnosed with an eating disorder following Anorexia Nervosa, Bulimia Nervosa, Eating Disorder Not Otherwise Specified and Binge Eating Disorder scored higher on ‘fussiness' than the un-exposed group.
		**4.2**.	**Personality**			
		4.2.1.	Anxiety trait	2	2, 26	Infants of mothers with higher anxiety traits showed negative emotionality and poor attention.
		4.2.2.	Alexithymic trait	1	6	Alexithymic trait in mothers, i.e., difficulty in identifying their feelings, predicted higher infant duration of orienting.
		4.2.3.	Perfectionistic trait	1	16	A higher level of adaptive perfectionism was negatively related to difficult temperament of the child.
**5**.	**Obstetric factors**	**5.1**.	**Fetal growth**			
		5.1.1.	Fetal growth	1	19	No significant relationship with fetal growth restriction.
		5.1.2.	Birth weight (overweight, underweight)	2	11, 22	A very small relationship with overweight at birth.
		**5.2**.	**Pregnancy complication**			
		5.2.1.	Gestational hypertension	1	12	Maternal hypertensive disorders were associated with difficult temperament.
		5.2.2.	Maternal nausea	1	31	Nausea in middle or late pregnancy was associated with lower sensory thresholds and higher level of activity and emotional intensity among infants.
		**5.3**.	**Delivery complication**			
		5.3.1.	Preterm delivery	1	34	Combination of preterm delivery and low birth weight was associated with difficult temperament.
		5.3.2.	Instrumental delivery	1	33	No significant relation between vacuum delivery and difficult temperament.
		5.3.3.	Artificial Reproductive Therapy	1	13	Mothers who received ART reported lower trait and state anxiety and infants had lower scores on difficult temperament.

*1. Number: Number of relevant articles, 2. Article Number: Article number referred to Appendix 2*.

#### Genetic and Biological Factors

Three studies identified the association between the genetic factors of infants and their temperaments (30, 37, 38). Torgersen and Kringlen (30) found that monozygotic (MZ) twins were more similar in temperament scores than dizygotic (DZ) twins, indicating that genetic factors may play a role in temperament (30). Wiebe et al. (37) claimed that the gene–environment (G-E) interaction between Taq*IA* polymorphism, which is related to the D2 dopamine receptor, and prenatal exposure to tobacco were observed for higher ‘attention' and ‘irritable reactivity' among 4-week old neonates (37). These authors concluded that the interplay between genetic and prenatal environmental factors might affect development patterns of the offspring. Ivorra et al. (38) reported that there were positive correlations between the *5-HTTLPR* gene of infants and the postnatal anxiety state of their mothers, and higher ‘irritability' among infants both at 2 and 8 months of age (38).

Out of the five studies that investigated the association of biological factors with infant temperament (14, 39–42), four measured stress hormones, such as maternal or placental cortisol, maternal corticotropin-releasing hormone (CRH), or adrenocorticotropic hormone (ACTH) (39–42), and one focused on foetal heart rate (FHR) (14). First, concerning stress hormones, most of the studies reported a significant correlation between increased levels of maternal stress hormones during pregnancy and difficult temperament in infants, such as higher levels of ‘emotional activity' (40) and higher levels of ‘fear and distress feeling', and ‘negative reactivity' (39, 42). Although most of these earlier studies hypothesised the role of maternal stress hormones as a mediator between maternal psychological distress during pregnancy and infant temperament (39, 40, 42), one study showed the insignificance of its mediating role using pathway analysis (41). Additionally, Davis et al. (39) measured placental cortisol and CRH in mothers' blood at 19, 25, and 31 gestational weeks, and found a significant association between both placental cortisol and CRH at 25 gestational weeks with higher ‘fear and distress feeling' among infants, but not at 19 or 31 gestational weeks (39).

Concerning FHR, Werner et al. (14) targeted 50 dyads of pregnant mothers and their unborn infants to assess FHR change. The authors found that foetuses during the third trimester of pregnancy who showed FHR increases during exposure to maternal psychological stress were more likely to exhibit higher ‘motor reactivity' in response to novelty at 4 months of age. However, Werner et al. (14) also reported no significant relationship between FHR change and ‘cry reactivity' of infants.

#### Environmental Factors

Six studies focused on maternal substance use during pregnancy; three assessed maternal smoking (37, 43, 44), one investigated paternal alcohol intake (45), and two measured maternal use of illegal drugs (46, 47).

The three studies that focused on maternal smoking reported that heavy or constant smoking by pregnant mothers predicted lower ‘positive mood' (43) and lower ‘reactivity to stimuli' (44) among infants, whereas Wiebe et al. (37) claimed that gene-environment interaction with exposure to tobacco caused higher ‘attention' and ‘irritability' as mentioned above. Picket et al. (43) also claimed that pregnant mothers who quit smoking early exerted a protective effect with decreased risk of ‘distress to novelty' and ‘irregularity' among infants. One study revealed that infants of alcoholic fathers displayed ‘stubborn' and ‘persistent temperament' at the age of 12 months (45). Edwards et al. (45) concluded that internalising problems are attributed to paternal depression comorbid to paternal alcoholism. Regarding maternal use of illegal drug, the use of illegal drugs was studied for cocaine or opiates (46), amphetamines, barbiturates, marijuana, and methamphetamine, as well as for alcohol (47). Locke et al. (46) reported that the infants of mothers who used cocaine or opiates scored lower in ‘smiling and laughter'. Weiss et al. (47) also concluded that infants, who were exposed to illegal substances during pregnancy, were more likely to be distracted by objects or events (‘poor attention to stimuli') in the environment.

#### Socio-Demographic Factors

Four studies investigated socio-demographic factors, namely low-income ethnic minorities and maternal nutrition (18, 48–50). Bush et al. (18) reported that prenatal stress caused by negative life events (NLE) among low-income women predicted greater respiratory sinus arrhythmia (RSA) reactivity and weaker recovery in foetuses, resulting in lower ‘surgency' and ‘regulation' of infants for weak effect but not negativity at the age of 6 months.

Chocolate consumption of mothers during pregnancy was related to prenatal stress; however, it was not associated with infant temperament (49). Brunst et al. (48) investigate the association between NLE, daily intake of polyunsaturated fatty acids (PUFAs) n3 and n6 during pregnancy, and infant temperament at 6 months postpartum. The authors found that higher n3:n6 ratios attenuated the effect of prenatal stress on ‘orienting' and ‘regulation' temperaments of infants in the African American population (48).

#### Psychological Factors

Seventeen articles investigated the association between prenatal mental health disorders of mothers and the temperament of infants longitudinally, nine focused on antenatal depression or anxiety in pregnancy (41, 50–57), five investigated prenatal stress (18, 48, 49, 58, 59), and one investigated eating disorders (60) and another investigated mindfulness (61). First, maternal antenatal depression and anxiety were assessed using the Structured Clinical Interview for the DSM (SCID) or parent-administered questionnaire. Six studies identified a significant association between maternal depressive or anxiety symptoms with ‘difficult temperament' in their child (41, 50–52, 56, 62). Antenatal depression in mothers was moderately associated with lower ‘affectivity' (41), lower ‘consolability' and higher ‘excessive crying' in their infants (56). Furthermore, one study suggested that a combination of maternal antenatal depression and lower family income was weakly associated with higher ‘emotionality and activity' among infants (50). The anxiety state of pregnant mothers was weakly associated with higher ‘emotional reactivity' (51, 52) and ‘poor attention to stimuli' among infants (51). However, three studies were unable to find an association between antenatal depression or anxiety in mothers and infant temperament (54, 57, 63).

Five articles focused on prenatal stress caused by severe stressful events (18, 48, 49, 58, 59). Contrary to the association between the combination of lower economic status and prenatal stress and *lower* ‘surgency' and ‘regulation' of infants as mentioned earlier (18), Lin et al. (59) reported that prenatal stress predicted *higher* ‘surgency' and ‘negativity', both of which directly and interactively predicted later engagement in regulatory behaviours. Laplante et al. (58) investigated the relationship between perceived maternal stress due to a disaster and infant temperament. Maternal distress was weakly associated with higher ‘fussiness' and ‘dullness' in infants. Raikonnen et al. (49) reported that ‘fear' in infants was higher for mothers who experienced severe stressful events prenatally than those who had not. One paper found a positive factor of infant temperament (61). Van den Heuvel et al. (61) found that mothers who were more mindful tended to score lower on self-regulation problems. Regarding eating disorders, infants of mothers who were diagnosed with an eating disorder such as Anorexia Nervosa (AN), Bulimia Nervosa (BN), Eating Disorder Not Otherwise Specified (EDNOS) and/or Binge Eating Disorder (BED), scored higher on ‘fussiness' than the un-exposed group (60).

The personality of mothers was studied in four articles. Two found that prenatal anxiety trait of mothers was associated with higher ‘negative emotionality' and ‘poor attentions' among infants (51, 57). One article targeting women with alexithymia (difficulty in identifying feelings) predicted higher infant duration for orienting (53). Another paper identified a higher level of adaptive perfectionism that was negatively related to difficult temperament in infants (64).

#### Obstetric Factors

Eight studies investigated an association between pregnancy or delivery complications and infant temperament (54, 55, 65–70). Concerning pregnancy complications, three studies assessed infants' weight at birth (54, 65, 66). Niegel et al. (65) found that a child with an overweight status at birth was more likely to show a difficult temperament (66). Baibazarova et al. (54) found that infants born with lower birth weight were more likely to show increased ‘fear' and ‘distress to limitation' (54). However, Roza et al. (64) concluded that there was no significant association between intrauterine growth and temperament after full adjustment (65). One study found that infants of mothers diagnosed with hypertensive disorders showed a difficult temperament for weak effect; however, the risk increased in the case of preeclampsia (70). Another study reported that nausea among mothers during pregnancy was associated with lower sensory thresholds and higher levels of activity and emotional intensity among infants (68). Ross (66) investigated the association between preterm delivery (highly correlated with lower birth weight) and difficult temperament (67). One study found no significant association between the mode of delivery (vacuum delivery) and infant temperament (69). Regarding pregnancy due to Artificial Reproductive Treatment (ART), mothers who received ART reported lower trait and state anxiety, and infants had *lower* scores on difficult temperament (55).

## Discussion

A variety of predisposing factors such as genetic, biological, environmental, socio-demographic, and psychological factors of parents were identified. Additionally, both pregnancy and delivery complications weakly or moderately affected infant temperament. Because of different study designs and statistical analysis methods in the reviewed papers, further studies are required to draw more accurate conclusions. However, this systematic review helps to clarify the overall picture concerning prenatal and intrapartum factors of infant temperament and its possible mechanisms.

Regarding the characteristics of the reviewed studies, different assessment tools for measuring infant temperament were used in the reviewed studies due to disagreements about the concept of temperament among researchers. As stated previously, the dimensions of temperament, the criteria of behavioural style, the relationship with emotional behaviour, relative stability, and inheritance were controversial among theorists (1). Therefore, different assessment tools were developed: IBQ, ICQ, CITQ, TTQ, and others. Associations between these temperament measures need to be studied in further investigations. Almost all the reviewed studies treated infant temperament as a dimension. However, children may be grouped according to the patterns of their temperaments. This is a shift of emphasis from variable-centred to person-centred approaches. Different types of children may show different associations with the predictors discussed in the review (71–76).

This systematic review found that only a few studies investigated outcomes during the neonatal period, whereas others assessed outcomes between 2 and 12 months of age. Although temperament is regarded as relatively stable during infancy, some specialists emphasised that the expression of temperament is changeable throughout the period. Rothbart mentioned that ‘bio-behavioural shift is marked during 2 to 3 months of age by increases in orienting, smiling, and laughter, but at a relatively stable activity level from 3 to 12 months' (10). Goldsmith also referred the temperament as primary emotions based on presumably neurophysiological underpinnings; however, some degree of preservation of rank order are present among individuals until the various facets of feeling states, action tendencies, and response systems become integrated into a functional system. Hence, this is the reason why only a few studies in this systematic review collected data in the neonatal period, as the expression of temperament may be unstable during this period.

How temperament is formed and affected by environmental factors during pregnancy is still unclear. Our systematic review revealed that infants with a certain genotype, such as TaqIA polymorphism or *5-HTTLPR* genes, who were prenatally exposed to tobacco (37) or psychological stress of their mothers (38), were more likely to show higher ‘irritability' and ‘fussiness'. The findings support the evidence that a child's temperament starts to form with a complex interaction of genes and biological reactions with the outer environment (G-E interactions) from the beginning of pregnancy (77).

Whether the environmental or psychological factors, including substance use or psychological distress, work as a modulator between gene and forms of temperament to affect directly or directly the development of temperament is also unclear. One research study showed two different trajectories through genetic factors as well as other factors of maternal stress hormonal activity called ‘biological embedding' (78). Influenced by the activation of the maternal HPA axis due to psychological/physical stress, the bio-physiologic functions of the foetus also change (79–81) and may result in a higher risk of infant negativity characterised by sadness, anger/frustration, fear, and poor soothability (39–42). Many of the reviewed studies included here focused on maternal psychological symptoms such as anxiety and depression during pregnancy and/or biomarkers of maternal stress such as cortisol, CRH, and ACTH. The increased levels of biomarkers, which are also closely linked with maternal psychological and physical symptoms, showed significant associations with difficult temperament such as higher emotionality, activity, lower surgency, poor attention, and higher levels of fear and fussiness. The biological response of increased stress hormone may play as a moderator interacting with psychological distress and temperament rather than a mediator (82). Additionally, recent studies have shown that psychological distress of mothers during pregnancy not only activates the foetal stress response directly but also indirectly causes an adverse intrauterine environment, such as pregnancy complications, including hypertensive disorders and foetal growth restriction, leading to negative consequences for infant temperament. Although our review did not cover posttraumatic stress disorders (PTSD), some previous studies indicated PTSD symptoms of mothers, cortisol, and difficult temperament of child (82, 83). Hence, ensuring mental and physical health of pregnant mothers is regarded as increasingly important by both clinical health professionals and researchers.

Moreover, it seems that substance use, such as the intake of tobacco, alcohol, and/or illegal drugs, by pregnant mothers directly impacts the neuropsychiatric development of their child because expressions of temperament are different depending on the substance used by the mother. For example, infants of mothers who had a habit of smoking showed higher ‘attention' and ‘irritability' (37), whereas infants of mothers who used cocaine or opiates scored lower on ‘smiling and laughter' and ‘poor attention to stimuli' (46). Due to the limited number of available articles, the differences in temperament due to the types of drugs are uncertain. Conversely, our review revealed the association between NLE, daily intake of PUFAs n3 and n6 during pregnancy, and infant temperament at 6 months postpartum, which supports the evidence that some kinds of nutrition buffer the adverse effect of psychological distress on the cognitive and neurological development (27, 28). However, considering mothers who are psychologically depressed, may take insufficient nutrition, the psychological distress may work as a confounder. Furthermore, whether *paternal* intake affects the temperament of the child is still unclear due to the very small number of studies (45). Further studies are required.

Interestingly, our systematic review identified that some significant obstetric factors were correlated with infants' temperament characteristics. They included foetal growth (birth weight), hypertension among mothers, nausea (emesis), and preterm birth. Further studies should confirm if low or high birth weight (66, 67) or if prematurity or low birth weight is important (67). Nausea (68) and dietary habits may also have an association with infant temperament. Furthermore, s other psychological and biological factors, the number of studies that focused on obstetric factors was insufficient; therefore, it is difficult to draw any conclusions from these results.

Some limitations should be noted. First, most of the reviewed studies adopted parent-administered questionnaires rather than objective measures to assess infant temperament. Although we excluded cross-sectional studies assessing psychological distress of parents and infant temperament simultaneously, perceived infant temperament may be biased due to parental mood at the time of observation (observer bias). Mothers with mental distress may have more negative views of parenting than others, such as a negligent attitude with decreased visual and physical contact with their infants (84, 85). Since depressed mothers are often more likely to perceive their child as difficult, caution should be taken regarding the causal relationship between infant temperament and parenting distress (8). Second, most of the significant factors identified in these longitudinal studies were only weakly or moderately associated with temperament. Considering the possibility that the expression of temperament may change along with social maturation in infancy (1), more underlying confounders in the postnatal period, such as bonding between the mother and infant, may affect the results. Third, the data search engines used were restricted to MEDLINE, PubMed, and SCOPUS because there was limited access to other databases in our institute. More published papers might have been obtained if we included other data search engines such as PsychINFO.

For future studies, study designs such as a prospective cohort study and adopting both subjective and objective measures may be recommended to avoid bias. Additionally, because most of the studies were conducted in Western societies targeting Caucasian children, more evidence needs to be accumulated in other ethnic groups because temperament is believed to be formed by genetic and biological functions. Furthermore, more evidence regarding pregnancy complications such as hypertensive disorders, nutrition status, delivery complications, and exposure to chemical factors (for example heavy metals, pesticides, and air pollutants) is needed to confirm the link with temperament (27).

Regardless of these limitations, this systematic review helps us understand prenatal and intrapartum factors of temperament, including genetic, biological, socio-demographic, psychological, and obstetric aspects in the complex mechanism of development. The findings from this systematic review imply the necessity of ensuring and promoting a safe and comfortable pregnancy and childbirth by health care professionals. Raising awareness of the risks of substance use and providing health education for a healthy pregnancy and childbirth to mothers and family members are recommended along with psychological support by nurses and midwives. However, it should be noted that infant temperament would, directly and indirectly, be associated with parent-child interactions as well as other confounders in childhood, subsequently helping to form their personality that would lead to positive or negative consequences later in their life (8). Hence, the expression of temperament may change flexibly, with positive consequences influenced by positive interactions between parent and child.

## Conclusions

This systematic review identified a variety of prenatal and intrapartum factors that are significantly associated with the temperament of infants: (1) genetic and biological factors: certain genotypes, maternal cortisol and ACTH, and CRHs, (2) environmental factors: substance use such as tobacco, alcohol, and illegal drugs, (3) socio-demographic factor: lower-income, (4) psychological factors: depression or anxiety, eating disorders, personality types of mothers, and domestic violence, and (5) obstetric factors: foetal growth (birth weight), hypertension in mothers, nausea (emesis), and preterm birth. These findings support the gene-environment interaction and biological mechanisms for developing infant temperament, suggesting the importance of ensuring a safe and comfortable environment for pregnant mothers, the unborn child, and families during pregnancy and delivery in society.

## Data Availability Statement

The original contributions presented in the study are included in the article/Supplementary Material, further inquiries can be directed to the corresponding author/s.

## Author Contributions

MTa contributed to the conception and designs of work, selection and review of articles, drafted and revised the manuscript. AM contributed to the selection of reviewed articles, drafted the manuscript. YO and TK contributed to the conception and designs of work, interpretation of data, supervision for drafting and revising the manuscript. MTo and LY contributed to supervision for drafting and revising the manuscript. All authors contributed to the article and approved the submitted version.

## Conflict of Interest

The authors declare that the research was conducted in the absence of any commercial or financial relationships that could be construed as a potential conflict of interest.
