# Supplementary material for: Prenatal and Intrapartum Factors Associated With Infant Temperament: A Systematic Review
Source: Front Psychiatry. 2021 Apr 8;12:609020. doi: 10.3389/fpsyt.2021.609020 (PMC8060501; doi:10.3389/fpsyt.2021.609020)
Supplement: Supplementary file 1 [file Data_Sheet_1.pdf]

Appendix 1. Inclusion, exclusion criteria and search strategy

**Inclusion criteria**	Original research article,<br><br>Article written in English<br><br>Article targeted to infant who is up to 12 months old or under
**Exclusion criteria**	Cross sectional studies investigated an association between postnatal mental status of parent and infant temperament.
**Search strategy (Pubmed, MEDLINE, Scopus)**	
#1	perinatal OR antenatal OR pregnancy
#2	(mental stress) OR (psychological stress) OR depression【Mesh】OR anxiety OR post traumatic stress【Mesh】
#3	((cocaine drug) OR (smoking) OR (alcohol) OR (drug) OR (cannabis))
#4	factor OR determinant OR predictor
#5	infant
#6	Temperament
#7	#1 AND #2 AND #5 AND #6
#8	#1 AND #3 AND #5 AND #6
#9	#1 AND #4 AND #5 AND #6
#10	#7 OR #8 OR #9

Appendix2. Summary results of selected articles

Article N	Authors	Year	Country	Design	Number	Assessment point	Predictors	Adjustment factors	Measure assessed temperament	Main findings
1	Bush, N. R., et al.	2017	USA	Longitudinal observation study	151	T1: 12 －24 gestational weeks, T2: 6 months postpartum	Stressful life events, perceived stress, low income, racial/ethnic minority	Gestational age, birth weight, poverty, prenatal perceived stress, postnatal perceived stress, postnatal perceived health	Infant Behaviour Questionnaire - Revised (IBQ-R)	Prenatal stress was associated with lower surgency (β = －0.23, $p < 0.05$) and regulation (β = － 0.33, $p < 0.01$).
2	Chong, S. C., et al.	2016	Singapore	Longitudinal observation study	1247	T1：26 gestational weeks T2: 3 months postpartum	Antenatal depression, anxiety trait and state	Age, ethnicity, marital status, household income, child care management	Early Infant Temperament Questionnaire (EITQ)	Maternal anxiety trait, state anxiety (p <0.001) were associated with negative emotionality and attentional regulation (anxiety trait β = 0.31, p = 0.05, anxiety state β = 0.42, $p < 0.001$) , whereas depression was not (p = 0.09).
3	Laplante, D. P., et al.	2016	Canada	Longitudinal observation study	121 (60 boys, 61 girls)	T1: During pregnancy T2: 6 months postpartum	Disaster-related maternal stress	Major life events, timing of exposure, maternal illness, objective hardship, subjective distress	Infant Characteristic Questionnaire (ICQ)	Maternal perceived stress explained infants fussy difficult temperament slightly (β = 0.04, p< 0.05).
4	Nolvi, S., et al.	2016	Finland	Longitudinal observation study	282	T1: 14, 24, and 34 gestational weeks, T2: 6 months postpartum	Depression, anxiety, pregnancy-specific anxiety	Maternal education, income, parity and civil status.	Infant Behaviour Questionnaire-Revised (IBQ-R). 'Negative reactivity' subscale	Pregnancy specific anxiety is associated with negative emotional reactivity (β = 0.20, $p < 0.001$), its aspects fearfulness (β = 0.15, p = 0.057), and falling reactivity (β = －0.22, $p < 0.001$).
5	Locke, R. L., et al.	2016	USA	Cohort study	366 (cocaine opposed) 37 (opiate exposed) 522 (matched comparisons)	4 months postpartum	Mother's history of admission due to cocaine or opioids intake during pregnancy	Age, ethnic group, marital status, economic status, prenatal care	Infant Behaviour Questionnaire (IBQ), Observation of infant's behaviour in reaction to stimuli.	Cocaine and opioid exposed infants scored lower on IBQ smiling and laughter. Infants with heavy cocaine exposure were more likely in high social negative reactivity profile (p < 0.05). Opiate-exposed infants were rated as less respondent to soothing($p < 0.05$).

Continued. Appendix 2

No	Authors	Year	Country	Design	Number	Assessment point	Predictors	Adjustment factors	Measure assessed temperament	Main findings
6	Kantonen, T., et al.	2015	Finland	Longitudinal observation study	102	T1: During pregnancy<br>T2: 6 months postpartum	Prenatal depression and pregnancy-related anxiety, alexithymic trait of mother	Maternal age, education, smoking in pregnancy, infant gender and gestational weight.	Infant Behaviour Questionnaire (IBQ)	Alexithymic traits of difficulty in identifying feelings predicted higher infant duration of orienting (β= 0.08, p < 0.001).
7	van den Heuvel, M., et al.	2015	Sweden	Longitudinal observation study	90	T1: Second trimester of pregnancy, T2: 10 months postpartum	Mindfulness state during pregnancy	Prenatal anxiety	Infant Behaviour Questionnaire-Revised Very Short From(IBQ-R.VSF)	Higher maternal mindfulness during pregnancy was negatively assessed with infant self-regulation problems (β = −0.25, p < 0.001) and less infant negative affectivity (β= −0.36, p < 0.001). Prenatal anxiety mediated between mindfulness and infant temperament.
8	Burnst, K.J., et al.	2014	USA	Cohort study	95(Western American), 53(African American), 107(Hispanic American)	6 months postpartum	Race, Negative life events during pregnancy, daily intakes of polyunsaturated fatty acids (PUFA)(n3, n6)	Maternal education, age, child's sex	Infant Behaviour Questionnaire (IBQ)	Prenatal stress effect on infant' orienting & regulation scores were modified by maternal n3: n6 ratios (p=0.03). As NLEs increased, lower n3:n6 ratios predicted lower infant orienting & regulation scores, whereas higher n3: n6 ratios attenuated the effect of prenatal stress.
9	Lin, B., et al.	2014	USA	Longitudinal observation study	77	T1: During pregnancy, T2: 6 week postpartum, T3: 12 week postpartum	Prenatal stress	Not assessed	Infant Behaviour Questionnaire Revised (IBQ-R)	In Structural Equation Modeling (SEM), prenatal stress predicts higher levels of infant negativity (β=0.22, p < 0.001) and surgency (β=0.22, p < 0.001). Both of which directly or interactively predict later engagement in regulatory behaviours.
10	Rouse, M. H., & Goodman, S. H.	2014	USA	Longitudinal observation study	77	T1: During pregnancy, T2: 3 months postpartum	Saliva cortisol, and major depression assessed by Structural Clinical Diagnosis (SCID).	Depression symptoms in other trimesters.	Infant Behaviour Questionnaire Revised (IBQ-R)	Antenatal depression is associated with negative affectivity (β=0.45, p < 0.001). No significant mediate role of maternal cortisol between antenatal depression and temperament of infants.

Continued. Appendix 2

No	Authors	Year	Country	Design	Number	Assessment point	Predictors	Adjustment factors	Measure assessed temperament	Main findings
11	Baibazarova, E., et al.	2013	UK	Longitudinal observation study	158	T1: 2nd trimester of pregnancy, T2: 3 months postpartum	Anxiety and birth weight	Maternal and placental cortisol	Infant Behaviour Questionnaire-Revised (IBQ-R) 'distress to limitations'	Lower birth weight is correlated with infant temperament (fear: $\beta = -0.16$, $p < 0.05$, distress to limitation: $\beta = -0.18$, $p < 0.05$) . However no significant relation between prenatal anxiety and infant temperament.
12	Robinson, M., et al.	2013	USA	Longitudinal observation study	2384	T1: During pregnancy, T2: 12 months postpartum	Hypertensive disorder during pregnancy	Maternal age, education, parity, pre-pregnancy maternal BMI, total family income, maternal smoking, the presence of the biological father in the family home, and the maternal stressful events, birth weight of child gestational age at birth, breastfeeding.	Australian revision of the Toddler Temperament Scale based on the Carey Infant Temperament Questionnaire	Maternal gestational hypertension increased the risk of difficult temperament of infants at 12 months (OR = 1.36) and preeclampsia increased the risk (OR = 2.17).
13	McMahon, C. A., et al.	2013	Australia	Cohort study	250 (ART), 262 (Comparison)	T1: Third trimester of pregnancy, T2: 2 month postpartum, T3: 4 month postpartum	ART, general anxiety, pregnancy focused anxiety,	Gestational weeks when mothers completed the anxiety assessment, child age, educational levels, marital status, gestational complications, prenatal depression. Smoking, alcohol intake.	Short Temperament Scale for Infants	Women conceiving through ART reported lower state and trait anxiety, but higher pregnancy-focused anxiety than their comparison groups ($p < 0.001$). Pregnancy-related anxiety is not associated with difficult temperament. ART women reporting less difficult infant temperament ($p < 0.001$).
14	Melchior, M., et al.	2012	France	Longitudinal observation study	1903	T1: 24 gestational weeks, T2: 12 months postpartum	Antenatal depression: CES-D, Family income.	Demographic factors: child age, sex, mother's age at birth, number of siblings, prenatal separation, and non-maternal care, premature birth, birth weight, duration of breastfeeding. Antenatal anxiety and postnatal depression	The EAS questionnaire	Combination of maternal antenatal depression and lower family income was associated with children's higher scores of emotionality ($\beta = 0.57$, $p < 0.001$).
15	Zerwas, S., et al.	2012	Norway	Cohort study	3013(Diagnosed of eating disorders), 45964(Comparison)	T1: 2nd trimester, T2: 6 months postpartum	Eating disorders: questionnaire based on DSM-Ⅳ	Smoking, primiparity, postpartum depression and anxiety, preterm birth and low birth weight	Infant Characteristic Questionnaire(ICQ) "Fussy/Difficult" subscale	Children of mothers diagnosed of eating disorder showed higher score of fussiness than un-exposure group. (AN: OR = 3.04, BN: OR = 1.94, EDNOS: OR = 4.12, BED: OR = 1.66)

Continued. Appendix 2.

No	Authors	Year	Country	Design	Number	Assessment point	Predictors	Adjustment factors	Measure assessed temperament	Main findings
16	Macedo, A., et al.	2011	Portugal	Longitudinal observation study	386	T1: 37 gestational weeks, T2: 3 months postpartum	Personality: Perfectionism (Multidimensional Perfectionism Scale; Self-oriented perfectionism (SOP), Socially Prescribed perfectionism (SPP), and Multidimensional Perfectionism Scale (MPS).	Depressive symptoms: BDI- II	Ad hoc questions	A higher levels of adaptive perfectionism (Self-oriented perfectionism) was negatively related to difficult child ($p < 0.05$).
17	Ivorra, J. L., et al.	2010	Spain	Longitudinal observation study	317	T1: 8 weeks postpartum, T2: 32 weeks postpartum	Molecular genotypes of child (5HTT, DRD4, and MAO-A functional polymorphisms).	Maternal depression and anxiety	Mother and Baby Scale (MABS)	The irritability scores of MABS showed significant relationship of mother's anxiety interacted with the 5HTTgenes ($\beta = 0.35$, $p = 0.001$).
18	Wiebe, S. A., et al.	2009	USA	Longitudinal observation study	98	T1: Before 28 gestation weeks, T2: 12, and 48 hrs of infant's delivery, T3: 4 week postpartum	Prenatal tobacco exposure, and gene environment interactions	Maternal education, sex of child.	Neonatal Temperament Assessment	In 4 week old neonates, genotype exposure interactions were observed for attention and irritable reactivity ($p < 0.01$).
19	Roza, S. J., et al.	2008	Netherland	Cohort study	7465	T1: 2nd and 3rd trimester, T2: 6 months postpartum	Fetal growth, IUGR	Socio-economic status (education, smoking, national origin), obstetric and neonatal factors (gestational age at birth, parity) and others (infant sex, infant age, maternal anxiety and depresion)	Infant Behavior Questionnaire - Revised (IBQ-R)	No significant association between Intra uterine growth restriction (IUGR) and infant's difficult temperament. After full adjustment, reduced fetal weight gain was only related to prolonged duration of orienting ($\beta = -0.38$, $p < 0.01$).
20	Pickett, K. E., et al.	2008	USA	Cohort study	18000	T1: 9 months postpartum	Smoking during pregnancy	Age, alcohol intake, depression history, partner perpetrated domestic violence, marital status, child sex, birth weight of child economic status	Carey Infant Temperament Questionnaire (CITQ)	Heavy or constant smoking during pregnancy were associated with low positive mood(OR = 1.17). Early quitting exerted a protective effect with decreased risk of distress to novelty (OR = 0.79) and irregularity (OR = 0.89).

Continued. Appendix 2

No	Authors	Year	Country	Design	Number	Assessment point	Predictors	Adjustment factors	Measure assessed temperament	Main findings
21	Davis, E.P., et al.	2007	USA	Longitudinal observation study	248	T1: 19 gestation weeks, T2: 25 gestation weeks, T3: 31 gestation weeks, T4: 2 months postpartum	Placenta cortisol	Postpartum depression and anxiety	Infant Behaviour Questionnaire (IBQ) 'fear' subscale	Placenta cortisol at 25 gestation weeks predicted temperament negative reactivity (β = 0.23, $p < 0.01$).
22	Niegel, S., et al.	2007	Norway	Cohort study	29182	T1: pregnancy, T2: 6m postpartum	Overweight, rapid early weight gain	Age, BMI pre-pregnancy, DM, Child sex	Infant Characteristic Questionnaire (ICQ)	Very small associations between fussy/difficult temperament and rapid weight gain during the first months of life during the first month postpartum (OR=1.06, 95%CI: 1.02-1.10).
23	Werner, E. A., et al.	2007	USA	Longitudinal observation study	50	T1: During pregnancy, T2: 4 months postpartum	Fetal heart rate	Psychiatric diagnoses by structured interview	Infant Behaviour Questionnaire (IBQ), Observed child reaction in the circumstance of novelty following Kagan *et al.*'s method	3rd trimester fetus who showed HR increases during exposure to maternal stress were more likely to exhibit high motor reactivity in response to the novelty (OR = 1.23). No significant relationship between FHRchange and infant cry reactivity or low reactive temperament.
24	Weiss, S. J., et al.	2007	USA	Longitudinal observation study	142	T1: During pregnancy, T2: 6 months postpartum	Prenatal exposure to illegal drug (alcohol, marijuana, or other drugs)by maternal report	Maternal stress, maltreatment	Revised Infant Temperament Questionnaire (RITQ)	Drug exposure accounted for 12 % of the variance in a child's likelihood of distractibility at the 6months of age (β= 0.36, $p < 0.001$).
25	Martin, R. P., et al.	2006	USA	Longitudinal observation study	6401	T1: 6 months postpartum, T2: 5 age, T3; 12 age	Maternal smoking during pregnancy	Psychiatric hospitalization, psychological distress during pregnancy, hospitalization for accidents, socioeconomic status, age, and symptoms of upper respiratory infection, nausea, child gender	Carey Infant Temperament Questionnaire (CITQ)	Infants of heavy smoking scored less stimulated and less biological irregular ($p <$ 0.05).

Continued Appendix 2.

No	Authors	Year	Country	Design	Number	Assessment point	Predictors	Adjustment factors	Measure assessed temperament	Main findings
26	Austin, M. P., et al.	2005	Australia	Longitudinal observation study	970	T1: Third trimester of pregnancy, T2: 4 to 6 months postpartum	Antenatal anxiety trait, depression, obstetric factors (emergency caesarean section)	Not assessed	The Short Infant Temperament Questionnaire (SITQ)	The antenatal trait STAI significantly predicted maternal report of difficult temperament at 4 or 6 months independent of postnatal depression scores (OR = 2.66). Antenatal depression and perceived life event stress were not predictive of temperament.
27	Davis, E. P., et al.	2005	USA	Longitudinal observation study	248	T1: 19 gestation weeks, T2: 25 gestation weeks, T3: 31 gestation weeks, T4: 2 months postpartum	Pregnancy corticotropin-releasing hormone (CRH) (Blood)	Postpartum depression and anxiety	Infant Behaviour Questionnaire-Revised (IBQ-R)	CRH at 25 gestation weeks was associated with infant temperament (fear and distress feeling) ($p < 0.05$) but not at 19 or 31 gestation weeks.
28	Räikkönen, K., et al.	2004	Finland	Longitudinal observation study	305	T1: pregnancy, T2: 6 months postpartum	Chocolate consumption during pregnancy, prenatal stress	Psychosocial stress experience	Infant Characteristic Questionnaire (ICQ)	Prenatal stress was associated with infant temperament. However chocolate consumption during pregnancy showed no significant relationship with infant's temperament ($p < 0.01$).
29	De Weerth, C., et al.	2003	Netherland	Longitudinal observation study	17	T1:3rd trimester, T2: 1w postpartum, T3: 3w postpartum, T4: 5w postpartum, T5: 7w postpartum, T6: 18w postpartum, T7: 20 w postpartum	Cortisol level (Saliva)	Not assessed	Infant Characteristic Questionnaire (ICQ), Behavioural observations taking an infant into bath.	A trend was found for the high cortisol infants to be delivered. In the categories, Emotion and Activity, the infants from the high prenatal cortisol levels during pregnancy had significantly higher scores than others ($p < 0.05$).
30	Edwards, E. P., et al.	2001	USA	Cohort study	94 (Paternal alcoholic), 17 (both alcoholic mother and father), 102 (comparisons)	T1: 12 months postpartum, T2: 18 months postpartum	Paternal alcoholism, antisocial behaviour, depression of parents, aggression of parents	Education, mother alcohol problems	Infant Characteristic Questionnaire (ICQ)	Infants of alcoholic father displayed more stubborn temperaments at age of 12m as univariate analysis (β = 0.22, $p < 0.05$) and more externalizing problems at 18 months (β = 0.15, $p < 0.05$). Internalizing problems are attributed to paternal depression cormobid to paternal alcoholism.

Continued Appendix 2.

No	Authors	Year	Country	Design	Number	Assessment point	Predictors	Adjustment factors	Measure assessed temperament	Main findings
31	Martin, R. P., et al.	1999	Finland	Longitudinal observation study	2948	T1: Second trimester, T2: Third trimester, T3: 6 months postpartum	Maternal nausea	General somatic and psychosomatic symptoms (depression, anxiety and stress)	Carey Infant Temperament Questionnaire (CITQ)	Children whose mothers reported nausea in middle or late pregnancy had lower sensory thresholds and higher levels of activity and emotional intensity in infancy(p < 0.05).
32	Zuckerman, B., et al.	1990	USA	Longitudinal observation study	1123	T1: During pregnancy, T2: Between 8 and 72 hours postpartum	Prenatal depressive symptoms	Ethnicity, age, gestational weeks, child gender, parity, substance use	Neurologic and Adaptive Capacity Scale	Prenatal depressive symptoms were associated with consolability and excessive crying of infant adjusting for sociodemographic factors.
33	Blennow, P. I., et al.	1988	Sweden	Cohort study	37 (Vaccuum extraction cases), 27 (Comparisons)	T1: 6 months postpartum, T2: 1 year postpartum, T3: 2 years postpartum	Vaccuum extraction method of delivery	Not assessed	Thomas and Chess's Temperament Questionnaire (TTQ)	Temperament was not systematically related to vaccum extraction delivery.
34	Ross, G.	1987	USA	Cohort study	98 (Preterm delivery), 89 (Comparisons)	T1: 12 months postpartum	Preterm delivery, infant development	Not assessed	Toddler Temperament Scale (TTS)	Preterm and full term infants did not differ significantly on overall temperament type. Poorer performance on the Bayley Mental Scales was significantly associated with temperament characteristics of lower adaptability (r = 0.34, p < 0.001), lower persistance (r = 0.29, p < 0.005), and more withdrawal from new stimulation (r = 0.31, p < 0.05).
35	Torgersen, A. M., et al.	1978	Norway	Longitudinal observation study	53	T1: 2 months postpartum, T2: 9 months postpartum	Genetic factors: MZ twin and DZ twin	Child's sex	Thomas and Chess's Temperament Questionnaire (TTQ)	Monozygotic (MZ) twins were more similar than dizygotic (DZ) twins statistically significant with regard to temperament (p < 0.05).

Appendix 3. Results of quality assessment

Article N.	Q1	Q2	Q3	Q4	Q5	Q6	Q7	Q8	Q9	Q10	Q11	Q12	Q13	Q14
1	Yes	Yes	Yes	Yes	No	Yes	Yes	Yes	Yes	Yes	No	No	Yes	Yes
2	Yes	Yes	Yes	No	No	Yes	Yes	Yes	Yes	Yes	Yes	No	No	Yes
3	Yes	Yes	Yes	Yes	No	Yes	Yes	Yes	Yes	Yes	Yes	No	No	Yes
4	Yes	Yes	Yes	Yes	No	Yes	Yes	Yes	Yes	Yes	Yes	No	No	Yes
5	Yes	Yes	Yes	Yes	No	Yes	Yes	Yes	Yes	Yes	Yes	No	No	Yes
6	Yes	Yes	Yes	Yes	No	Yes	Yes	Yes	Yes	Yes	Yes	No	No	Yes
7	Yes	Yes	Yes	Yes	No	Yes	Yes	Yes	Yes	Yes	Yes	No	No	Yes
8	Yes	Yes	Unknown	Yes	No	Yes	Yes	Yes	Yes	Yes	Yes	No	Yes	Yes
9	Yes	Yes	Yes	Yes	No	Yes	Yes	Yes	Yes	No	Yes	No	Yes	Yes
10	Yes	Yes	Yes	Yes	No	Yes	Yes	Yes	Yes	No	Yes	No	Yes	Yes
11	Yes	Yes	Yes	Yes	No	Yes	Yes	Yes	Yes	No	Yes	No	Yes	Yes
12	Yes	Yes	Yes	Yes	No	Yes	Yes	Yes	Yes	Yes	Yes	No	No	Yes
13	Yes	Yes	Yes	Yes	No	Yes	Yes	Yes	Yes	Yes	Yes	No	No	Yes
14	Yes	Yes	Yes	Yes	No	Yes	Yes	Yes	Yes	Yes	Yes	No	No	Yes
15	Yes	Yes	Yes	Yes	No	Yes	Yes	Yes	Yes	Yes	Yes	No	No	Yes
16	Yes	Yes	Unknown	Yes	No	Yes	Yes	Yes	Yes	Yes	Yes	No	No	Yes
17	Yes	Yes	Yes	Yes	No	Yes	Yes	Yes	Yes	No	Yes	No	No	Yes
18	Yes	Yes	Unknown	Yes	No	Yes	Yes	Yes	Yes	No	Yes	No	No	Yes
19	Yes	Yes	Yes	Yes	No	Yes	Yes	Yes	Yes	Yes	Yes	No	No	Yes
20	Yes	Yes	Yes	Yes	No	Yes	Yes	Yes	Yes	Yes	Yes	No	Yes	Yes
21	Yes	Yes	Yes	Yes	No	Yes	Yes	Yes	Yes	Yes	Yes	No	No	Yes
22	Yes	Yes	Yes	Yes	No	Yes	Yes	Yes	Yes	Yes	Yes	No	No	Yes
23	Yes	Yes	Yes	Yes	No	Yes	Yes	Yes	Yes	No	Yes	No	No	Yes
24	Yes	Yes	Yes	Yes	No	Yes	Yes	Yes	Yes	No	Yes	No	No	Yes
25	Yes	Yes	Unknown	Yes	No	Yes	Yes	Yes	Yes	No	Yes	No	No	Yes
26	Yes	Yes	Unknown	Yes	No	Yes	Yes	Yes	Yes	No	Yes	No	No	Yes
27	Yes	Yes	Unknown	Yes	No	Yes	Yes	Yes	Yes	Yes	Yes	No	No	Yes
28	Yes	Yes	Unknown	Yes	No	Yes	Yes	Yes	Yes	Yes	Yes	No	No	Yes
29	Yes	Yes	Unknown	Yes	No	Yes	Yes	Yes	Yes	Yes	Yes	No	No	Yes

Continued. Appendix 3.

Article N.	Q1	Q2	Q3	Q4	Q5	Q6	Q7	Q8	Q9	Q10	Q11	Q12	Q13	Q14
30	Yes	Yes	Unknown	Yes	No	Yes	Yes	Yes	Yes	No	Yes	No	No	Yes
31	Yes	Yes	Unknown	Yes	No	Yes	Yes	Yes	Yes	No	Yes	No	No	Yes
32	Yes	Yes	Unknown	Yes	No	Yes	Yes	Yes	Yes	No	Yes	No	No	Yes
33	Yes	Yes	Yes	Yes	No	Yes	Yes	Yes	Yes	No	Yes	No	No	Yes
34	Yes	Yes	Unknown	Yes	No	Yes	Yes	Yes	Yes	No	Yes	No	No	Yes
35	Yes	Yes	Unknown	Yes	No	Yes	Yes	Yes	Yes	No	Yes	No	No	Yes

NIH Study Quality Assessment Tool

Q1. Was the research question or objective in this paper clearly stated?

Q2. Was the study population clearly specified and defined?

Q3. Was the participation rate of eligible persons at least 50%?

Q4. Were all the subjects selected or recruited from the same or similar populations (including the same time period)? Were inclusion and exclusion criteria for being in the study prespecified and applied uniformly to all participants?

Q5. Was a sample size justification, power description, or variance and effect estimates provided?

Q6. For the analyses in this paper, were the exposure(s) of interest measured prior to the outcome(s) being measured?

Q7. Was the timeframe sufficient so that one could reasonably expect to see an association between exposure and outcome if it existed?

Q8. For exposures that can vary in amount or level, did the study examine different levels of the exposure as related to the outcome (e.g., categories of exposure, or exposure measured as continuous variable)?

Q9. Were the exposure measures (independent variables) clearly defined, valid, reliable, and implemented consistently across all study participants?

Q10. Was the exposure(s) assessed more than once over time?

Q11. Were the outcome measures (dependent variables) clearly defined, valid, reliable, and implemented consistently across all study participants?

Q12. Were the outcome assessors blinded to the exposure status of participants?

Q13. Was loss to follow-up after baseline 20% or less?

Q14. Were key potential confounding variables measured and adjusted statistically for their impact on the relationship between exposure(s) and outcome(s)?
